# Can’t Count or Won’t Count? Embedding Quantitative Methods in Substantive Sociology Curricula: A Quasi-Experiment

**DOI:** 10.1177/0038038515587652

**Published:** 2015-06-29

**Authors:** Malcolm Williams, Luke Sloan, Sin Yi Cheung, Carole Sutton, Sebastian Stevens, Libby Runham

**Affiliations:** Cardiff University, UK; Cardiff University, UK; Cardiff University, UK; Plymouth University, UK; Plymouth University, UK; Cardiff University, UK

**Keywords:** embedding, pedagogy, quantitative methods, quasi-experiments, teaching

## Abstract

This paper reports on a quasi-experiment in which quantitative methods (QM) are embedded within a substantive sociology module. Through measuring student attitudes before and after the intervention alongside control group comparisons, we illustrate the impact that embedding has on the student experience. Our findings are complex and even contradictory. Whilst the experimental group were less likely to be distrustful of statistics and appreciate how QM inform social research, they were also less confident about their statistical abilities, suggesting that through ‘doing’ quantitative sociology the experimental group are exposed to the intricacies of method and their optimism about their own abilities is challenged. We conclude that embedding QM in a single substantive module is not a ‘magic bullet’ and that a wider programme of content and assessment diversification across the curriculum is preferential.

## Introduction: The Quantitative Deficit in British Sociology

The quantitative deficit in British Sociology (and social science more generally) has become a matter of wide concern, debate and more recently the focus of significant investment in resources (Adeney and Carey, 2011; MacInnes, 2009; Payne and Williams, 2011; Taylor and Scott, 2011; Williams et al., 2004, 2008). What causes the ‘deficit’ and how it might be tackled are not settled issues, though its effects can be seen in the paucity of reported quantitative research in sociology (MacInnes, 2009; Platt, 2012; Williams et al., 2004) and in the low proportion of quantitatively focused PhD projects and Masters/undergraduate dissertations. The latter have implications, not just for the discipline, but for sociologists^1^ entering the labour force more generally. The focus of the present paper is on one particular diagnosis: that the separation of the teaching of quantitative methods from its substantive subject context is a problem (though certainly not the only one) and it is consequently hypothesised that the uniting of quantitative methods teaching with that context will produce beneficial results. We report here on a recent ‘quasi-experiment’ to embed quantitative methods teaching in two Year Two undergraduate degree programmes in UK universities (University A in Wales and University B in England). The research aimed to assess the effectiveness of this strategy as measured by changes in attitudes toward learning quantitative methods before and after the experiment (as compared to those students who did not participate) and improvements in student awareness and confidence about quantitative methods. Surprisingly, to the authors’ knowledge, this is the first time in the UK that a formal programme of embedding methods in a sociology module and a subsequent evaluation of the outcomes have been attempted. The article is organised as follows:

In the first section, we briefly review the ‘quantitative’ problem and the reasons for conducting the experiment. In the second section, we discuss the context, the methods and the limitations of the research. In the third and principal section, we compare the findings between the control group and the experimental group, before and after the intervention, in respect of their attitudes and their self-reported abilities.

## What is the Problem?

That there *is* a problem of some kind is a consensual position, though sociologists, and social scientists more generally, will emphasise different aspects of the nature and extent of the problem. Some have focused on the structure and career pathways of the discipline and thus the quantitative deficiency of its teachers (Platt, 2012), more broadly the ‘anti scientific’ nature of the discipline (Williams, 2000), or simply on the numeric deficit, often seen to have its origins in secondary – even primary – education (Porkess, 2011). This is certainly the position of the Royal Statistical Society, who, with their Centre for Statistical Education (RSSCSE) and GETSTATS programme, have done much to promote the effective learning of statistics (and improved numeracy) in schools and postsecondary education. This work is not wholly confined to social science and the emphasis in social science (and specifically sociology) has been on promoting the effective teaching of quantitative methods more generally. More recently the position statement *Society Counts*, led by the British Academy (2012) and signed by several professional bodies that support academic social science, has attempted to specify the issues in clearer terms. Moreover, the problem is not entirely confined to the UK and is a particular problem in most of the countries of the Anglophone world, with the exception of the USA (see, for example, Parker, 2011).

Though not the first work in this area, research conducted in the early 2000s (Williams et al., 2004) indicated the somewhat ‘Cinderella’ status of quantitative methods teaching. In effect, quantitative methods modules existed in a ghetto, seen by faculty staff not involved in their teaching as a necessary evil. The Sociology Benchmark criteria of 2007 (QAA, 2007c) briefly mentioned quantitative methods, but they did not prescribe how, or how much, quantitative methods should be taught (see below). Indeed, in an exploratory study of sociology undergraduate teaching units undertaken in 2002, 74% of undergraduate sociology degrees dedicated more than 5% of content to quantitative methods and in 26% of degrees this was more than 11% (Williams et al., 2004). Additionally, in this study, over 60 quantitative methods teachers, at various career stages, were brought together for two one-day consultation events in Edinburgh and London. These were used to draw out what practitioners saw as the issues underlying the ‘quantitative problem’ (Williams et al., 2004: 20–28). The key emerging themes were the isolation of quantitative methods teaching staff who were often junior colleagues, ‘briefing against’ quantitative methods by other staff as irrelevant or even antipathetic to good social ‘science’, and more generally the separation of quantitative methods from the rest of the curriculum, evidenced by the very small number of students who used quantitative evidence in other work, most particularly in their dissertations.

These findings were borne out in a national review of quantitative methods teaching by John MacInnes in 2009 (MacInnes, 2009). A national study of sociology student attitudes to quantitative methods (conducted in 2006/07) confirmed an overall negative attitude and some lack of confidence in learning quantitative methods (Williams et al., 2008). However, this was not overwhelmingly a ‘maths’ problem. Fifty per cent reported a good experience of maths at school (as compared to 43% who had a bad experience). The large size of the minority is important, of course, because it both represents stored up problems that may manifest later at university and will inevitably show up as important effects in subsequent research. Similarly, slightly more students saw themselves as good at maths than those who saw themselves as bad at it (44% and 42%, respectively). Seventy-five per cent disagreed with the statement: ‘One of the reasons I chose this degree is because I don’t like maths’. Conversely, 64% reported a preference for writing an essay, rather than conducting data analysis. However, the key finding of this study was that (whatever their views) there was a major lacunae in the training of sociology undergraduates. For example, knowledge of data analysis techniques was rudimentary. Less than 50% of students studying research methods learn about Z-tests, 20% had learned about Pearson’s *r*, 31% about chi-square, 45% about regression and only 70% of students had undertaken any quantitative analysis (using SPSS and/or Minitab). The overall picture can be said to add up to a lack of understanding of what a quantitative approach might comprise and would certainly indicate an inability of students to critically appreciate quantitative evidence presented in articles or monographs.

Although this study provided detailed evidence, it was more confirmatory than surprising and on the basis of what was already widely perceived to be a problem. A number of initiatives in the mid-2000s, many funded through the UK Economic and Social Research Council (ESRC), were aimed at tackling various aspects of the problem. For example, strategies were employed to persuade, or induce students, to carry out their dissertations using quantitative methods, to make more use of secondary datasets and the pooling and sharing of electronic resources (see papers in Payne and Williams (2011) for accounts of many of these). Despite this the problem persisted and the implications of a weak undergraduate quantitative base were recognised by the ESRC, leading to both the appointment of a Strategic Advisor (Professor John MacInnes) for the teaching of quantitative methods and two funding initiatives in 2012 (Curriculum Innovation and Researcher Development Initiative). Twenty projects were funded (including the current one). At the time of writing, a consortium of the Nuffield Foundation, ESRC, the Higher Education Funding Council for England and the British Academy intend setting up around 15 UK centres of excellence in quantitative pedagogy with the intention of bringing about a ‘step change’ in the way quantitative methods are taught and in the number of quantitatively competent social science graduates (Nuffield Foundation, 2013). The problem, or problems, are yet to be resolved, but there is a widespread awareness and concern about their existence that is translating into substantial resources aimed at their resolution.

So what is the problem or problems? The research so far suggests three likely issues. The first is lack of ability in or fear of numbers, but though certainly a key issue it does not account for all of the student negativity/antipathy toward quantitative methods. A second problem might be to do with the nature of current professional sociology in the UK, which itself is overwhelmingly qualitative, where any systematic research methods are used and are ‘humanistically’ focused (Payne et al., 2004).^2^ The A level syllabi places very little emphasis on quantitative methods and it is possible for students to mostly avoid assessment in this area. In most schools and colleges, sociology is within a ‘humanities’ pathway and few students combine sociology with either maths or a natural science.^3^ Consequently, students who come to university have expectations of sociology as a discipline of critique (in the manner of cultural studies), which is then reinforced by the teaching they receive at university. In the national study of student attitudes in 2006/07, 71% of respondents regarded sociology as closer to the arts and humanities than science; this, combined with a preference for essay writing over data analysis and a less than clear cut finding on numerical ability, suggests more of an attitudinal problem. This finding was explored further in focus group interviews, which overwhelmingly indicated a humanistic inclination and antipathy toward quantitative methods (Williams et al., 2008).

Which brings us to the third problem: that the style of teaching in sociology separates out ‘knowing’ from ‘doing’ (perhaps unsurprising given that a large proportion of UK sociology output does not employ empirical methods) (see Williams et al., 2004). UK sociology, though not unique in its style of undergraduate teaching methods, typically offers, in the first two years, a portfolio of modules in social issues and problems including gender, sexuality, identity, religion, etc., social theory modules which teach syllabi either around topics (e.g. agency-structure) or around theorists (classical and contemporary), and combined research methods modules. In both University A and University B there was very little empirical content in substantive modules, beyond illustrative references to studies conducted. Certainly there was no ‘hands on’ empirical content and this would seem to be a typical way of teaching sociology in the UK. The 2004 study found that 86% of sociology courses taught at least some quantitative methods within mixed methods modules and 30% of courses taught more qualitative than quantitative methods (Williams et al., 2004). In the third year, students will specialise and take classes often in those areas of staff research specialisms (e.g. ethnicity, gender, work and employment, youth and identity, media, etc.). Sometimes limited specialist modules are offered in year two, but on the whole (with the exception of methods modules) students do not ‘do’ sociology, they learn about others doing sociology and because the majority of UK sociology is qualitatively focused (Payne et al., 2004), their exposure to doing quantitative sociology is limited to tasks undertaken in generic research methods modules. This is not universally the case (Williams et al., 2004), but it is mostly so.

This marks sociology out from virtually all science disciplines and even many arts disciplines, where lab time and studio work is considered a crucial component of learning. Sociology students lack experiential learning. The single exception to this comes in the final year when most students undertake a dissertation project. In both of the universities, which are the focus of the current study, less than 10% use quantitative methods (either primary or secondary data) in their dissertations.^4^ If the position found in the 2004 study has not substantially changed, the research methods teaching in the second year is the only formal empirical training in their discipline that most undergraduate sociology students get. Though, as we noted above, in many universities there has been some investment in teaching resources and anecdotally it would seem several courses have introduced some element of methods revision in the third year. Nevertheless, the experiential learning that sociologists receive is mostly at the end of their degrees and only through consultative supervision (i.e. it is expected they will already possess most of the necessary skills to successfully carry out dissertation work).

Contrast this with the forms of teaching and learning in biology where students are expected to ‘undertake sufficient practical work to ensure competence in the basic experimental skills’, ‘undertake field and/or laboratory investigations of living systems’ and ‘obtain, record, collate and analyse data in the field and/or laboratory’ (Bioscience Benchmarks; QAA, 2007a). Similarly, the Chemistry Benchmarks aim to ‘develop in students the ability to apply standard methodology to the solution of problems in chemistry’ or ‘the ability to interpret and explain the limits of accuracy of their own experimental data in terms of significance and underlying theory’ (Chemistry Benchmarks; QAA, 2007b). In contrast, the Sociology Benchmarks are replete with terms such as ‘critical awareness’, ‘self-reflection’ and ‘understanding’, and in the matter of practical skills little is said beyond: ‘the ability to identify a range of qualitative and quantitative research strategies and methods and to comment on their relative advantages and disadvantages’, or ‘the ability to conduct sociological research in a preliminary way’ (Sociology Benchmarks; QAA, 2007c).

Sociologists are taught to be critical (in the relativistic sense) and reflective consumers of mostly qualitative or theoretical materials, rather than analytic sociological practitioners. One might say that the non-empirical teaching methods and the benchmarks are mutually reinforcing, in that the latter reflected current practices and the former continued them through adherence to the benchmarks.

## Methods

The experiment, or at least its most rigorous variant the randomised control trial (RCT), has been described as a methodological ‘gold standard’ in behavioural research in that it is the best method to capture causality (Bonell et al., 2011). The logic is simple and elegant. If the level, or the amount, of an independent variable is changed and there is a subsequent change in the dependent variable, then that change must have resulted from the change in the independent variable. In a controlled laboratory setting such an aspiration is realistic, but in the open systems of social life a simple cause–effect sequence is hard to establish. In RCTs a randomised sample from the same population is divided into an ‘experimental group’ and a ‘control group’, with the former receiving some or other treatment. Devices such as ‘blinding’ or placebos are used to prevent behavioural ‘contamination’ across the groups. In most social settings RCTs are very difficult to set up and indeed are themselves methodologically not beyond reproach (Bonell et al., 2011; Byrne, 2011: 48–49).

Experimental methods in both education and sociology have a long history, particularly in the USA. The work of Donald Campbell was seminal in establishing the experiment as a legitimate research strategy in the evaluation of social and education programmes in the USA (Cook and Campbell, 1979). These were often large-scale social programmes in areas such as income maintenance, housing subsidies, prisoner rehabilitation programmes, educational performance, etc. (Oakley, 2000: 198–230). Mostly these experiments randomised participants into control and experimental groups and (for its period) used quite sophisticated modelling techniques. However, the results, though often valuable in informing knowledge of context and policies, rarely produced unequivocal results and this apparent failure has often been cited since as evidence of their lack of efficacy or precision. Yet these social experiments were often with small samples and posed more complex questions than equivalent clinical trials. A key factor in successful experiments is inevitably sample size, yet, as Ann Oakley notes (2000: 233), critics have often (in small sample research) confused *no evidence of effect* with *evidence of no effect*, wrongly inferring the latter from the former.

In social experiments, particularly those in education, randomisation is also difficult to achieve. Consequently, ‘quasi’-experiments have been used for many years in a variety of settings such as public health (Petticrew et al., 2005) and community safety (Bennet, 1988). The logic of the manipulation of independent variable(s) and ‘experimental’ and ‘control’ groups are retained and there is not always an expectation of an unequivocal outcome. Indeed, realists, such as Ray Pawson and Nick Tilley, have noted in respect of evaluation research, that outcomes are a product of complex mechanisms operating in contexts (Pawson and Tilley, 1997). It follows that whilst a mechanism may be present in both contexts A and B, only in A will the mechanism operate. Realist evaluations place little emphasis on knowledge accrued from one-shot evaluations and hold that firmer conclusions about mechanisms ensue from the accumulation of knowledge in different contexts (Pawson, 2006). Rarely are single experiments ‘crucial’, in the Popperian sense of falsifying a null hypothesis, and can often only be evaluated alongside a number of similar interventions or through a systematic review (see, for example, Petticrew and Roberts, 2006). While one can establish temporal precedence easily enough, and despite its potential in educational research, in a quasi-experiment there is always a threat to internal validity where one cannot rule out plausible alternative rival explanations (Cook and Campbell, 1979).

The current research might be seen as being in the same tradition of classroom interventions as those pioneered in the US Head Start interventions (Oakley, 2000: 227) and it is indeed a ‘one-shot’ experiment, but hopefully one from which contextual or counterfactual findings might be explored in further research.

The research described here was conducted across one academic year (2012/13) in two universities (A and B). Both universities have long-established sociology and social science courses and both take an interdisciplinary approach to teaching. In both universities the second level research methods module constituted the control group and was compulsory for all students, including those in the experimental group. The ‘experimental’ modules were also in Year Two and were well established, though were redesigned to ‘embed’ quantitative content. Students taking the experimental modules opted to do so voluntarily. In this sense, there was informed consent, though the ‘quantitative’ content was neither hidden nor emphasised and the module descriptors provided details of what would be taught.

The research can be summarised as at least a partial operationalisation of the question: ‘What would happen if sociologists were taught more like scientists (or indeed artists)?’. In practice, and more specifically, it aimed to do the following:

- create one module at each institution which embeds QM in a substantive area;
- run a quasi-experiment in which a group of students at each institution study the embedded module at Year Two;
- compare and contrast knowledge of and attitudes towards QM after the learning experience with students who did not take the embedded module; and
- evaluate the impact of ‘embedding’ on student knowledge and attitudes towards QM.

An ideal and unlikely result of our research would have been a very clear pattern of improved attitudes towards and awareness of quantitative methods in the experimental group, but such a clear-cut result is unlikely for a number of reasons. First, there were important and complex changes in both the experimental *and* control groups, between time *t*^1^ and *t*^2^ (they are complex systems, in the sense meant by Pawson and Tilley). The interventions in the former were substantial, but the latter also studied a generic research methods module, in which (as we note above) there had been significant pedagogic investment. Moreover, it would be anticipated that all students would develop educationally through virtually a whole year of their degree. For the experimental module a whole range of quantitative/statistical skills were introduced, some particular to the intervention modules (over-sampling and weighting), though at a basic level with the technicality of calculating the actual weights. Others involved further or more detailed work on those learnt in the generic methods modules (which all students took). For example, students moved on from two-way simple cross-tabulations to three-way tables. They critically read and interpret statistical outputs from three-way cross-tabulations using real-world (and often dirty) data from complex large data sets. Assessments include the evaluation of competing theories’ ability to best explain the observed empirical patterns. Students also discussed the nature of missing data and the implication they have on the kind of conclusions drawn. Similarly, the attitudes and awareness comprised the measurement of over 100 items. Finally, a single classroom-based experiment will inevitably have a relatively small number of participants. Thus the experiment in the current research is very much a complex intervention combining a large number of measures with a small *n* sample. The effects, or indeed their absence, across a number of measures requires a nuanced interpretation as a first attempt to compare embedded quantitative methods teaching with standard approaches.

The research utilised both quantitative and qualitative methods, but were based around comparing competencies and attitudes, both before and after the intervention, in the experimental and control groups. The principle instrument for this was an online questionnaire, but supplemented with focus group interviews and observations of classes. The questionnaire replicated a number of measures used in the 2006/07 ‘national’ study of sociology student attitudes, in order to permit a comparison of results (Williams et al., 2008).

## Embedding Quantitative Methods in Sociology Modules

The learning outcomes for the modules where quantitative methods were embedded give a flavour for what was taught:

- evaluate statistical significance of results and the relationship to sample sizes;
- understand the links between theory, evidence and evaluation;
- identify and utilise secondary data sources and evaluate their usefulness;
- understand the gaps in secondary data and identify the need for primary research;
- understand the concept of weighting in sample survey;
- understand the methodological basis of alternative research designs;
- develop statistical analysis skills appropriate for the level of study; and
- develop appropriate written and verbal communication skills for quantitative methods.

We selected two Stage 2 modules for the embedding exercise: ‘Migration, Race and Ethnic Relations’ at University A and ‘Knowing the Social World’ at University B. Enrolment is broadly the same, with 48 (University A) and 42 students (University B). Quantitative materials were introduced primarily as seminar exercises. Skills in interpreting quantitative results were taught and practised. Students worked in small groups in advance and during the seminar, which provided a ‘safety in numbers’ comfort factor in case they got it wrong.

While the statistical knowledge primarily ‘tracked’ what was taught in the generic Research Methods module, additional concepts such as weighting and statistical control (by introducing a third variable) were also introduced. Students also learned how to collect and analyse different kinds of observational data: survey data including Census 2011 and ethnographic data based on a neighbourhood observation exercise. Seminar exercises were unassessed but students were required to write-up two short papers based on one of the seminar topics, combining the data analysis and theoretical perspectives covered in lectures. Data analysis questions also appeared in exams where students were tested on how to interpret statistical results rather than memorising facts and formulae.

Overall, the structured approach for seminar exercises provided a clearer framework for discussion. Even students who turned up unprepared had something to say using statistical findings provided during the session. This in turn reduced the frequent ‘mini lectures’ delivered by the tutor. Students also welcomed the combination of the ONS 2011 Census interactive maps and the neighbourhood observation exercise.

Despite the large investment and effort, the embedding experiment had not won students over hands down. Against an environment in which assessment is predominantly essay based, students were pushed beyond their ‘comfort zone’. Even though clear guidelines were given, there has been considerable anxiety of writing short seminar reports and end of term papers instead of essays.

The response rates at *t*^1^ and *t*^2^ in each group are given in Table 1.

**Table 1.** Control and experimental responses by institution at *t*^1^ and *t*^2^.

	University B		University A	
	Experimental (*n*)	Control (*n*)	Experimental (*n*)	Control (*n*)
*t*^1^ (Oct 2012)	11	133	34	159
*t*^2^ (April/May 2013)	2	24	16	82

Regrettably the low baseline number of students in the experimental group at University B was diminished to such an extent at *t*^2^ that the students from this institution had to be dropped from the analysis. A high drop-out rate for respondents is likely due to the fact that the ‘experiment’ was not conducted in a controlled environment and we were reliant on goodwill for survey completion.^5^ Whilst the loss of an inter-institutional comparison is disappointing, we continue with the University A data alone.

## Experimental and Control Baseline Comparison

To check for selection effects we tested for significant differences in attainment, experiences and attitudes between the control and experimental groups. There were no significant differences in the GCSE maths grade, retrospective overall percentage mark for the first year of the degree programme, subjects studied before coming to university or UCAS (Universities and Colleges Admissions Service) tariff points, confirming that there were no academic or pre-university selection effects. However, there was a difference in attitudes that may have been influenced during the first year of university and the experimental group were significantly more confident in using numbers in everyday life than the control group (means of 73.09 and 65.10, respectively, on a 0–100 scale; *t* = 1.97, 190 d.f., *p* = 0.05). The experimental group were also significantly more inclined to see their main degree subject as closer to science/maths than the control group (means of 4.68 and 4.01, respectively, on a nine-point scale where 1 = closest to arts/humanities and 9 = closest to science/maths; *t* = 1.94, 191 d.f., *p* = 0.05). Further statistically significant differences at the *p* = 0.05 level relating to knowledge of, and perceived difficulty of, quantitative concepts are summarised in Table 2.

**Table 2.** Significant differences in responses between control and experimental groups.

Item		Experimental response	Control response
Have you studied …	Ratios	Yes = 84.8% (*n=28*)	Yes = 97.5% (*n=153*)
		No = 15.2% (*n=5*)	No = 2.5% (*n=4*)
	Validity	Yes = 70.6% (*n=24*)	Yes = 90.4% (*n=141*)
		No = 29.4% (*n=10*)	No = 9.6% (*n=25*)
	Reliability	Yes = 73.5% (*n=25*)	Yes = 93.7% (*n=148*)
		No = 26.5% (*n=9*)	No = 6.3% (*n=10*)
How difficult do you find … *(where 1 = easy and 5 = hard)*	Odds	1 = 39.3% (*n=11*)	1 = 23.5% (*n=31*)
		2 = 10.7% (*n=3*)	2 = 39.4% (*n=52*)
		3 = 39.3% (*n=11*)	3 = 22.0% (*n=29*)
		4 = 10.7% (*n=3*)	4 = 9.8% (*n=13*)
		5 = 0.0% (*n=0*)	5 = 5.3% (*n=7*)
	Frequencies	1 = 35.5% (*n=11*)	1 = 12.8% (*n=18*)
		2 = 29.0% (*n=9*)	2 = 34.0% (*n=48*)
		3 = 25.8% (*n=8*)	3 = 31.9% (*n=45*)
		4 = 6.5% (*n=2*)	4 = 15.6% (*n=22*)
		5 = 3.2% (*n=1*)	5 = 5.7% (*n=8*)
	Standard deviation	1 = 13.8% (*n=4*)	1 = 2.3% (*n=3*)
		2 = 10.3% (*n=3*)	2 = 22.7% (*n=30*)
		3 = 31.0% (*n=9*)	3 = 27.3% (*n=36*)
		4 = 27.6% (*n=8*)	4 = 28.8% (*n=38*)
		5 = 17.2% (*n=5*)	5 = 18.9% (*n=25*)

There are clearly some differences between the groups, which is not unusual in a quasi-experiment (Petticrew et al., 2005); however, there are also 93 survey item measures where the groups did not differ significantly, thus indicating an overall high level of similarity.

## Measuring *t*^1^ to *t*^2^ Attitudinal Change

Table 3 illustrates changes in attitudes between *t*^1^ and *t*^2^ for the experimental and control groups. Students were asked whether they agreed, disagreed or were unsure of the statements listed on the left-hand side of the table and we have presented the proportion of those ‘agreed’ and those ‘unsure’ alongside the percentage point change in agreement between the two time points. Values for each measure have been calculated only for respondents with responses at both waves (case-wise deletion).

**Table 3.** Changes in attitudes between *t*^1^and *t*^2^ for the experimental and control groups.

	*t* ^1^				*t* ^2^				% Change (agree)	
	Experimental (%)		Control (%)		Experimental (%)		Control (%)		Experimental	Control
	*agree*	*unsure*	*agree*	*unsure*	*agree*	*unsure*	*agree*	*unsure*		
In my university work I would rather write an essay than use statistics	50.0	18.8	70.0	16.3	56.3	12.5	61.3	18.8	+6.3	−8.7
Statistics are for geeks	0.0	6.3	6.3	2.8	0.0	0.0	5.0	8.8	0	−1.3
On the whole you can’t trust statistics	25.0	12.5	13.8	25.0	6.3	31.3	8.8	23.8	−18.7	−5.0
I feel confident about learning statistics	56.3	0.0	46.3	26.6	43.8	25.0	49.5	24.1	−12.5	+3.2
Good numeric skills will help me get a job	87.5	6.3	82.5	6.3	81.3	12.5	68.8	18.8	−6.2	−13.7
I don’t think social science students should have to study statistics	6.3	18.8	2.5	12.5	18.8	12.5	10.0	12.5	+12.5	+7.5
Statistical evidence is important when considering sociological theory	87.5	6.3	75.9	21.5	68.8	12.5	77.2	17.7	−18.7	+1.3
Understanding statistics helps me to understand social research	75.0	6.3	86.3	7.5	87.5	12.5	78.8	8.8	+12.5	−7.5
You don’t need to use quantitative data in order to understand sociological phenomena	18.8	18.8	20.3	20.3	31.3	12.5	29.1	16.5	+12.5	+8.8

The results paint a mixed picture, which is not always positive. In favour of the embedded curriculum model we can observe that the experimental group are less likely to be distrustful of statistics than the control group, although the latter also saw a modest increase in levels of trust. After the intervention the experimental group are also more positive about the role of statistics in understanding social research compared to the control group, which has become more negative. However, there are several measures where the intervention appears to have had a negative effect on student attitudes such as a preference for essays over statistics in assessment, confidence about learning statistics and the relevance of statistics in understanding sociological theory. For these three items the control group are more positive at *t*^2^ than *t*^1^, demonstrating that this is not a result of engagement with quantitative methods in the wider curriculum. Despite these differences there are universal trends that cut across groups, albeit to different magnitudes, such as a decrease in agreement over the importance of numeracy in employability, whether social science students should study statistics and whether quantitative data are needed to understand sociological phenomena. These latter findings are truly puzzling, though it seems unlikely that these attitudes would result from more exposure to quantitative methods teaching, other than the articulation of a resistance to being exposed to quantitative work, which is perceived by many as difficult.

## Measuring *t*^1^ to *t*^2^ Perceived Difficulty Change

Table 4 shows the changes in perceived difficulty of concepts and techniques for the experimental and control groups between *t*^1^ and *t*^2^. Respondents were asked to rate each item on a five-point scale, where 1 = easy and 5 = hard, and we present the arithmetic mean of each group at the two time points and the standard deviation. The two columns at the right-hand side of the table measure change in attitudes and a negative value indicates that a concept was perceived as easier at *t*^2^ than at *t*^1^. Significant results have been identified using the Wilcoxon signed rank test, which allows us to test whether the mean ranks differ between *t*^1^ and *t*^2^ (Bryman and Cramer, 2011); thus, we are testing for significant changes in attitudes between time points within the experimental and control groups. Values for each variable have been calculated only for respondents with responses at both waves (case-wise deletion).

**Table 4.** Changes in mean perceived difficulty between *t*^1^and *t*^2^ for the experimental and control groups.

	*t* ^1^		*t* ^2^		Change	
Difficulty scores *(where 1 = easy and 5 = hard)*	Experimental	Control	Experimental	Control	Experimental	Control
	Mean (s.d.)	Mean (s.d.)	Mean (s.d.)	Mean (s.d.)		
Averages	1.81 (0.98)	1.68 (0.84)	1.44 (0.72)	1.54 (0.82)	−0.37*	−0.14
Mean	1.88 (1.26)	1.49 (0.77)	1.13 (0.34)	1.32 (0.60)	−0.75**	−0.17
Median	1.69 (0.95)	1.46 (0.79)	1.13 (0.34)	1.38 (0.59)	−0.56**	−0.08
Mode	1.56 (0.97)	1.56 (0.93)	1.19 (0.40)	1.33 (0.63)	−0.37	−0.23**
Ratios	2.13 (1.15)	2.20 (1.11)	2.31 (1.14)	2.06 (1.01)	+0.18	−0.14
Odds	1.73 (1.01)	2.29 (1.12)	1.82 (0.87)	2.20 (1.02)	+0.09	−0.09
Percentages	1.69 (0.79)	1.94 (1.03)	1.56 (0.89)	1.69 (0.92)	−0.13	−0.25**
Probability	2.07 (1.10)	2.00 (0.98)	2.27 (1.22)	1.90 (1.01)	+0.20	−0.10
Trends	2.17 (1.34)	1.96 (0.90)	2.17 (1.03)	1.91 (0.99)	+0.00	−0.05
Statistical significance	2.67 (1.23)	2.98 (1.27)	2.42 (0.79)	2.31 (1.15)	−0.25	−0.67***
Correlation	2.25 (1.18)	2.09 (0.95)	1.94 (0.93)	1.83 (0.97)	−0.31	−0.26**
Chi-square	3.15 (1.41)	3.68 (1.29)	2.77 (1.09)	3.06 (1.13)	−0.38	−0.62***
Cross-tabulations	4.43 (0.98)	4.29 (1.21)	2.71 (0.76)	3.07 (1.09)	−1.72**	−1.22***
Frequencies	2.07 (1.16)	2.47 (1.11)	2.00 (1.07)	2.03 (1.04)	−0.07	−0.44***
Bar charts	1.33 (0.60)	1.30 (0.55)	1.31 (0.87)	1.38 (0.67)	−0.02	+0.08
Pie charts	1.69 (0.79)	1.61 (0.78)	1.19 (0.54)	1.42 (0.68)	−0.50***	−0.19*
Boxplots	3.00 (1.54)	2.78 (1.48)	2.17 (0.94)	2.40 (1.28)	−0.83	−0.38*
Line graphs	2.00 (1.10)	1.64 (0.73)	1.63 (0.81)	1.67 (0.91)	−0.37	+0.03
Scatterplots	2.29 (1.33)	2.02 (1.10)	1.29 (0.61)	1.62 (0.76)	−1.00***	−0.40***
Standard deviation	3.50 (1.16)	3.17 (1.09)	2.57 (0.94)	2.72 (1.15)	−0.93*	−0.45***
Variance	3.30 (1.34)	2.86 (1.19)	3.10 (0.57)	2.59 (1.13)	−0.20	−0.27
Validity	2.36 (1.28)	2.38 (1.04)	1.93 (1.07)	2.15 (1.03)	−0.43	−0.23*
Reliability	2.29 (1.14)	2.15 (0.85)	2.07 (1.00)	2.03 (1.00)	−0.22	−0.12
Induction	3.10 (1.45)	3.24 (0.94)	2.80 (1.23)	2.73 (1.18)	−0.30	−0.51**
Deduction	2.92 (1.56)	3.13 (0.95)	2.42 (1.17)	2.77 (1.25)	−0.50*	−0.36*
Operationalisation	2.80 (1.69)	2.56 (1.18)	2.70 (1.25)	2.53 (1.40)	−0.10	−0.03
Hypothesis testing	2.71 (1.27)	2.54 (0.95)	2.29 (1.07)	2.13 (0.99)	−0.42	−0.41**

* *p* < 0.1, **p < 0.05, ***p < 0.01.

s. d.: standard deviation.

It is striking that all significant shifts in difficulty scores reveal that students perceived the items to be easier at *t*^2^ than at *t*^1^, demonstrating that, despite the increase in negativity towards quantitative methods explored in the previous section, students are actually learning and finding the concepts easier to grasp and employ. This is in contrast to previous studies that have suggested that a high proportion of students’ dislike of numbers is related to the fact that they find it difficult (Williams et al., 2008).

By far the biggest perceived reduction in difficulty for both the control and experimental groups was for cross-tabulations, which is encouraging considering that they are the workhorse of social science quantitative analysis as most social survey data are categorical. The reduction in difficulty was notably higher than for any other item for the experimental group and just less than twice that of any other item for the control group. Whilst there are five items in which both groups show significant changes in perceived difficulty (cross-tabulations, pie charts, scatterplots, standard deviation, deduction), there are several changes that are specific to the experimental and control. Being subject to the intervention appears to be associated with students finding some concepts significantly easier than at the start of the year, including averages, means and medians, compared to the control group. However, not being part of the experimental group seems to be of greater benefit, with significant decreases in perceived difficulty for eight items, including the mode, percentages, statistical significance, correlation, chi-square, frequencies, boxplots, validity and induction. There are three potential reasons for this strange occurrence (strange on the basis that all of these students sat the same research methods modules). First, some of the shifts in scores for the experimental group may not be significant due to the low *n*, thus reducing confidence levels. When looking at the magnitude of the changes for the items where the control group shows a significant shift and the experimental group does not, the mean change values are not always small. Second, we know that there were baseline differences between the two groups and the tables suggests that those who were subject to the intervention found certain concepts notably easier than their counterparts at *t*^1^, such as percentages, statistical significance, chi-square, frequencies and induction. Third, we cannot ignore the possibility that participation in the experimental module could be detrimental to student experience of quantitative methods. We develop this last point in the next section.

## Conclusion

This was a small experiment over a limited time frame using only one cohort of students. It was essentially exploratory in nature and teaching staff themselves were teaching embedded methods with undergraduates for the first time. Students who opted to take the modules did not do so on the basis of their enhanced quantitative content, but equally there was little dissatisfaction with the embedded curriculum, and module evaluations produced results in line with most other modules in the students’ courses.

The findings were complex and even contradictory.

There were some improvements between time *t*^1^ and *t*^2^ in the experimental group, who were notably less likely to be distrustful of statistics than the control group; the experimental group were also more positive about the role of statistics in understanding social research compared to the control group, which became more negative in attitude. Yet, against this, the experimental group had an increased preference for essay writing over statistical work and were less confident about their statistical abilities.

Changes in perceived ability were even more complex, with improvements over time in both control and experimental groups. Those in the experimental group did report less difficulty in some items, after the intervention, but this may be due to a selection effect, in so far as those in the experimental group reported at *t*^1^ that they found some concepts easier than did those in the control group.

We cannot rule out the possibility that embedding quantitative materials in substantive modules can have a negative impact on student learning and perception of numbers. That, once exposed to a rigorous diet of ‘doing’ sociology, specifically the emphasis on using empirical data to warrant arguments, they find that it is hard and their optimism about their own abilities is tested. This, of course, is a worst case scenario. A less pessimistic interpretation is that students swapped some unknown unknowns, for some known unknowns. Prior to taking the module, they were unaware of their limited ability. At the time of the experiment this was the only module with embedded quantitative content. However, with quantitative content embedded in more modules issues of confidence and perception should become clearer – for better or worse!

Even if the first of these scenarios was the case, it is not an argument for abandoning embedding, but rather indicates the need to do other things alongside other approaches. For example, using a wide range of embedding strategies and materials to differing extent across many modules, not just quantitative but also qualitative, and/or tailoring generic methods modules toward practical tasks and reinforcing methods learning through repetition across the three years of an undergraduate course.

The findings of this study should not lead us to reject embedding, but rather to take a more measured and nuanced approach toward them and how we evaluate them. Sociology students are not biology or fine arts students and they have different expectations of their courses, likely shaped by their A level experiences in many cases. Abolishing generic methods modules in favour of embedding is not a magic bullet, but an increase in substantively embedded modules and a wider diversification of assessment to avoid stigmatisation of such ventures may pay dividends. Moreover, more comparative studies such as the present one, in different contexts and over a longer period, should begin to show a clearer picture of what works for whom in which circumstances.

## Notes

1. Though the current paper focuses on sociology, the problem, the ways it has been tackled and the results show little difference across analogous disciplines, such as criminology, social policy, health studies or politics. Indeed, in most British undergraduate methods courses, students from these disciplines are taught together. In this article the ‘experimental modules’ were sociology modules, but taken by students from the above disciplines. The ‘control’ group were equally heterogeneous.

2. In the study of output in major UK sociology journals (Payne et al., 2004), it was found that nearly 40% of published papers did not use any empirical methods.

3. Examples can be found at www.catscollege.com/en/courses/alevel/ or www.ccb.ac.uk/public/courses/parttime/access-to-higher-education-diploma-humanities-and-social-sciences-sept-12-6159.html (accessed 10 October 2013).

4. University A holds an undergraduate dissertation conference each year. In analysis of abstracts, in 2013, where methods are specified, two-thirds cited ‘semi structured depth interviews’ as their data collection method.

5. University B operates a three-term timetable. Term 3, after Easter, is assessment only and the absence of students on site reduced the possibilities for reminders/follow up. However, to check for systematic non-response, we compared students at University A who responded at *t*^1^ only against those who responded at *t*^1^ and *t*^2^. We found no significant differences in gender, GCSE maths grades, and perceptions of whether the social sciences are closer to the arts and humanities or science and maths. There was also little difference in UCAS points (grouped in 100s). Prior to the experiment, focus groups were conducted in each university. Despite the rather different modules taken, there was a consistency of attitudes toward quantitative methods, in these groups, from each university and, indeed, findings were rather similar to the findings from the focus groups in an earlier study of sociology students in English and Welsh universities (Williams et al., 2008).
